# Caries of the Right Parietal Bone

**Published:** 1886-11

**Authors:** Augustus V. Park

**Affiliations:** Chicago, Member of the American Medical Association, etc.; 411 Twenty-sixth Street


### ABSTRACT OF A REPORT OF A CASE OF CARIES OF THE RIGHT PARIETAL BONE, CAUSED BY A RAILWAY INJURY. *By* AUGUSTUS V. PARK, M.D., *Chicago, Member of the American Medical Association, etc.*

[Read before the Chicago Medical Society September 6.]

The case I have to present this evening is that of Edward M., aged 16 years, of medium height, stout, healthy and well nourished, by occupation a book-keeper. The family history on the father's side is good; the mother is now in the insane asylum at Jefferson. On the evening of September 18, 1883, while riding home from work, he attempted to cross the railroad track at the Twenty-sixth street crossing, but was compelled to wait until a long out-going freight train had passed. He then started to drive across the track, and at this moment his horse was struck and killed, instantly, by an incoming express train which was running at a high rate of speed, over the Grand Trunk Railway's tracks. The young man was found one hundred feet down the track, and north and east of where the horse was killed. He was carried from the place to a little cottage near the track, and I saw him in a few minutes. I found him unconscious, with pupils widely dilated, the pulse hardly perceptible, feeble and intermittent, breathing slow, and drawn at intervals. The features were ghastly and the face cold and pale. I examined the more extensive wounds and controlled the hæmorrhage. He was removed to his home, at Twenty-fifth street and Prairie avenue. There I made a careful and very thorough examination. His clothing, which was torn in shreds, was removed, and the following wounds were found: A contused and lacerated wound three inches wide on the back, two inches to the right of spinal column, and extending from the posterior superior spine of the ilium upward to the spinous process of the seventh dorsal vertebra. Lacerated and contused wounds were found above and below each elbow, and a large contusion on the left gluteal muscle. There was injury to the coccyx, causing long and continued coccygodynia, and the lower extremities were bruised in many places. A contusion existed on the right side of the forehead, extending from the orbital arch upward three inches. There was a V-shaped wound over the left ear, and, also, a large semi-circular wound on the top of the head, nearly in the centre of the right parietal bone. This wound, as well as the one over the left ear, extended through the scalp, the pericranium being stripped off in places. The wounds about the head and face were filled with sand and dirt. The scalp-wound was thoroughly cleansed, and the cranial wall carefully examined for fracture. The edges were then approximated as nearly as possible and closed with interrupted silk sutures. All the other wounds were cleansed and dressed with iodoform and carbolized cosmoline. The pupils of both eyes continued widely dilated, the left larger than the right. There was no vomiting or hæmorrhage from mouth or nose or ears. The shock was so great that it resembled collapse.

Twelve hours after the accident I found the patient still unconscious ; his temperature was 100° F., pulse 90, respiration 20. The wounds were all redressed. The tissues about the head and face were œdematous and the eye-lids puffy and closed.

Twenty-nine hours after the accident, his temperature was 102° F., pulse 112, respiration 20, and his face and scalp were becoming more and more œdematous. The patient was semi-unconscious, restless, and passed his urine voluntarily. I gave a hydragogue cathartic and quinine in three grain doses combined with iron. A lotion of the acetate of lead was used externally.

Forty-four hours after the accident, his temperature was 103.50° F., his pulse 120, and his respiration 20 to 22. He was delirious, and the condition of the face and scalp was unchanged. The wounds about his head and face were secreting but slightly.

Professor Daniel T. Nelson, their family physician, saw the boy in consultation with me and made a careful examination of the wounds of the head, and particularly the cut above the left ear and the one on the top of the head. He could not detect a fracture, or depression of the cranial wall, and advised that drainage-tubes be introduced. This was done and the dressings were re-applied as before. The quinine and iron were continued and the lotion applied externally as before.

The railway company's surgeon saw the boy with me and was permitted to examine the wounds for fracture, but with negative results. As regards treatment, he had nothing to suggest. The face and scalp became more and more œdematous ; the lips, nose and ears were greatly enlarged ; the eyes entirely closed, and the face of a light purple color.

The symptoms subjectively and objectively at this time, and for the next fourteen days, remained nearly the same. I will state, however, that the highest temperature observed was 103.8° F. There was but slight discharge through the drainage-tubes for two or three days, and they were removed on the fourth day after insertion. When the boy commenced to regain consciousness memory was greatly impaired and he could not distinguish one person from another.

On October 4, seventeen days after injury, his temperature was 99° F., his pulse 90, and respiration 18, the œdematous condition of the face was subsiding, the ecchymosis fading away and patient, by making an effort, could partially open the eyes. He complained of pain on the top of his head, and the dressings were removed and the wound examined. Several small pockets of pus were found. They were broken down with a probe and the wound washed with a two per centum carbolized solution and dressings applied as usual; all the other wounds had commenced to close by granulation.

On November 26, seventy days after the accident, I detected and detached a piece of exfoliated bone, the entire thickness of the external plate, and the size of a three cent piece. This piece of bone was removed through a fistulous canal which led down to it. The dressings were applied twice daily, yet the wound refused to close up as all the others had done, and denuded bone could be felt with the probe. I advised an immediate operation for the removal of more of the dead bone if present. To this his father would not consent, owing to the danger of erysipelas attacking the patient after the operation.

On December 17, ninety-two days from the day of the injury, at my request, Professor Daniel T. Nelson again saw the patient with me, and we decided to operate. At three o'clock P.M., the patient was brought to my office. I was assisted by Professors Daniel T. Nelson and R. W. Bishop. Mr. Hugo von Hermonn, Mr. Shoeneman, Mr. Meyer were present. A grooved director was inserted into the fistulous canal, and carried down to the cranial wall and the scalp slit up. This incision was then lengthened by a free cut, two inches down to the diseased bone, and the scalp and pericranium, which were thickened and hypertrophied, were drawn apart. Hæmorrhage was free, owing to the great vascularity of the part. The cranial wall was found in a carious condition over a much larger area than we expected to find. I was compelled to enlarge the incision in each direction before all of the diseased bone could be reached. This condition extended through the external table and I removed with a curette the diseased bony tissue down to the diploe, over an oval shaped area two inches and a half in width by three and a half in length. The hæmorrhage from the diploic veins was not great.

On December 18, at eight o'clock A.M., his temperature was 101° F., his pulse 90, and respiration 18. The patient complained of severe pain in the back and sides of the neck and of headache, and there was slight evidence of approaching facial erysipelas. The dressings were removed and the discharge was free and the drainage as complete as could be desired.

On December 19, his temperature was 103° F., his pulse

110, and respiration 22. He still complained of severe pain in his head and neck, with a burning sensation over the entire face and scalp. His eyelids were again closed and puffy. On removal of the dressings a partial suspension of the secretion from the wound, and a complete arrest of the adhesive process were found. Medication for the erysipelatous complication was continued.

On December 21, at eight o'clock A. M., his temperature was 100° F., his pulse 90, and respiration 18. The patient seemed to be improving in every way. His face and scalp were less œdematous, and the wound had commenced to slough to such an extent that some of the stitches were washed away with the carbolized solution. We now had a gaping wound an inch in width by four inches in length, which was cleansed, and its edges approximated as nearly as possible and held in position by compresses and bandages.

On December 25, the patient was without fever or pain. His pulse and respiration were normal. The wound was secreting healthy pus and the granulations had a healthy appearance, and the patient was taken to his home.

On February 4, one hundred and fifty-one days after the accident and forty-nine days following the removal of carious bone, the patient was discharged and the result obtained is quite as good as could reasonably be expected. Much was accomplished in this case in preventing a large and unsightly cicatrix by keeping up a series of carefully applied bandages and compresses. None of the compresses were applied directly over the wound, but in such a manner as would approximate the granulating edges.

411 Twenty-sixth Street.

